# Exogenous ketone therapy does not protect brain tissue after moderate-sized intracerebral hemorrhage despite signs of early neurological benefit

**DOI:** 10.1371/journal.pone.0311778

**Published:** 2024-12-05

**Authors:** Noam H. Sander, Shubham Soni, Cassandra M. Wilkinson, Elmira Khiabani, Jason R. B. Dyck, Frederick Colbourne

**Affiliations:** 1 Neuroscience and Mental Health Institute, University of Alberta, Edmonton, Alberta, Canada; 2 Cardiovascular Research Centre, University of Alberta, Edmonton, Alberta, Canada; 3 Department of Pediatrics, Faculty of Medicine & Dentistry, University of Alberta, Edmonton, Alberta, Canada; 4 Department of Psychology, University of Alberta, Edmonton, Alberta, Canada; Stanford University, UNITED STATES OF AMERICA

## Abstract

Ketone bodies, or ketones, are an alternative energy source and have several nonmetabolic signaling actions, such as inhibiting inflammation. Because of this, exogenous ketone supplementation has been used to help treat various diseases. β-hydroxybutyrate (βHB) is the major ketone body that has reduced neurological injury and brain edema in animal models of ischemic stroke and traumatic brain injury. However, the therapeutic potential of βHB in intracerebral hemorrhage (ICH) has not yet been determined. Here we investigated the effects of exogenous βHB treatment following ICH on inflammation, edema, injury size, and functional outcomes. To do this, we administered 250 mg/kg of βHB (subcutaneously every 12 hours) starting 2 hours after collagenase-induced ICH in rats over 3 experiments. First, we observed that βHB-treated rats had significant reductions in transcript expression of pro-inflammatory markers *Il1b* (p = 0.0210), *Tnfa* (p = 0.0108), and *Mcp1* (p = 0.0473) 3 days post-ICH. Second, βHB significantly improved neurological deficits measured by the neurological deficit scale on day 3 (p = 0.0416) in another cohort of rats, despite no treatment effect on edema (p = 0.2110). To test whether the effects of acute βHB treatment (for 7 days post-ICH) were chronically sustained, the third experiment used serial behavioural testing which confirmed that βHB significantly improved neurological deficit scores (p = 0.0459) 3 days post-ICH. These effects were not sustained at 7, 14, and 28 days post-ICH (all p≥0.1546). Similarly, βHB treatment did not yield differences in forelimb use asymmetry (all p>0.45) or brain lesion volume (p = 0.3381), the primary endpoint of this study. Thus, our studies show that an acute βHB treatment post-ICH can provide some early signs of functional benefit without evidence of lasting effects or neuroprotection. However, it remains to be tested whether other βHB dosing regimens may favorably affect these and other neurological, behavioral, and biochemical parameters, particularly given the early signals of reduced striatal inflammation.

## Introduction

Intracerebral hemorrhage (ICH) is a subtype of stroke characterized by bleeding in the brain that can have devastating outcomes, and accounts for about 10–20% of all strokes. The initial injury after ICH is directly caused by the mechanical trauma of blood quickly extravasated into brain tissue [1]. This is followed by secondary injury arising from various mechanisms, including excitotoxicity, neurotoxicity, inflammation, and brain edema [1–3]. Secondary injury develops over days to weeks after ICH [2, 4], making it an ideal cytoprotective goal that has been successfully targeted in preclinical studies [5, 6], but not yet in a clinical setting. As such, there remains a need for new therapies which can mitigate the direct and secondary injury that occurs from ICH.

The inflammatory response is one of the many important aspects which contributes to injury following ICH. Inflammatory cells, such as microglia, macrophages, and lymphocytes, are quickly activated and recruited to the perihematomal area and contribute to edema development and ultimately secondary brain injury [7]. Several factors, such as inflammatory cell activity, contribute to the injury pathways in ICH, including the release of pro-inflammatory cytokines such as interleukin (IL)-1β, IL-6, and tumor necrosis factor α (TNFα) [8]. This inflammation contributes to brain edema which develops in the perihematomal area within 24 hours and peaks approximately 3 days after ICH in rodents [9]. Perihematomal edema is a common endpoint in preclinical research [10] as it is thought to be causally important in determining functional outcomes and to be a surrogate marker of inflammation and cell death [11]. Edema can increase intracranial pressure and cause death, but importantly, it can be resolved if there is a reduction in pro-inflammatory signal and augmentation of restorative inflammatory responses (e.g., to aid in clearance of the hematoma) [12, 13]. Thus, targeting inflammation can be an important approach to reducing neurological injury in ICH.

One important pathway involved in inflammation and injury development is the NOD-like receptor pyrin domain containing 3 (NLRP3) inflammasome, a network of proteins which forms to allow for the production and release of IL-1β [14]. The NLRP3 inflammasome mediates inflammation in traumatic brain injury (TBI), ischemic stroke, and ICH, and NLRP3 inhibition reduces inflammation and improves outcomes in all these models of neurological injury [15–18]. However, while the currently available NLRP3 inhibitors are not safe for human use, other clinically translatable approaches are needed; one important approach to consider is the use of ketone therapy.

Ketones, namely β-hydroxybutyrate (βHB), are known to be metabolic molecules which are endogenously produced from fatty-acid oxidation during carbohydrate deprivation [19]. However, ketones are recognized to have important signaling functions which can contribute to improving health and disease [20]. Most notably, βHB is known to directly inhibit the NLRP3 inflammasome to reduce IL-1β production and elicit functional benefits [21]. Thus, ketones may serve as a therapeutic approach to reducing the injury and sequalae of ICH. Ketone therapy has been investigated in animal models of acute brain injury. Specifically, elevating βHB levels (endogenously or exogenously) has provided both neuroprotective and behavioural benefits in models of acute brain injury such as TBI and focal ischemia [22–29]. However, no studies to date have assessed whether βHB can be neuroprotective in ICH. Given the similarities in the pathophysiological events that contribute to brain injury, ketone therapy may be of particular interest as a therapeutic approach for ICH.

Herein, we investigated exogenous ketone therapy in rats after collagenase-induced ICH, a common model that causes bleeding for over several hours upon insult [4, 30]. The collagenase model has been well characterized with an inflammatory response, edema development, and behavioural deficits [4, 31]. Due to the various types of ketone therapies used in animal models (e.g., ketogenic diet, fasting, exogenous ketone administration), it is difficult to compare doses across studies. We gave 250 mg/kg of βHB (subcutaneous) as similar doses in animal models of other diseases have shown effects in the central nervous system [32, 33]. First, we assessed the effect of exogenous βHB treatment on inflammation in the acute phase of ICH by quantifying perihematomal inflammatory transcript expression, as well as neurological function using the Neurological Deficit Scale (NDS). We hypothesized that βHB treatment starting 2 hours post-ICH and given twice daily would reduce markers of inflammation and improve neurological deficits at 3 days post-ICH. Second, we tested the short-term effects of our ketone therapy on perihematomal edema and further investigated neurological function. We predicted that βHB treatment would attenuate perihematomal edema and improve neurological deficits 3 days post-ICH. Although inflammation and edema peak around 3 days after ICH in rodent models, they continue causing cell death for weeks [4, 8]. Thus, we extended our βHB treatment to 7 days after the initial insult and assessed total injury size and functional outcomes up to 28 days in our third experiment with the hypothesis that βHB treatment would reduce brain injury and improve long-term functional outcomes.

## Methods

This study is reported in accordance with the Animal Research: Reporting of *In Vivo* Experiments (ARRIVE) guidelines [34]. Experimental procedures were all performed in accordance with the Canadian Council on Animal Care guidelines and approved by the Biosciences Animal Care and Use Committee at the University of Alberta (AUP960).

Experiment 1 was an exploratory study, for which not all endpoints were pre-planned. Based on the results, experiment 2 and 3 were planned *a priori* to improve translational rigor. A total of 73 male Sprague Dawley rats were obtained from Charles River Laboratories (Saint Constant, Quebec). All animals weighed between 300 and 550 g (∼3–5 months old) at the time of ICH induction. To control for any between-group biases, all animals were exposed to the same conditions and behavioural tests, and kept in temperature and humidity-controlled rooms with a constant light cycle (12 hours light, 12 hours dark). All experimental procedures were performed during the light phase, except for half of the vehicle (VEH) and βHB injections. Food (Purina rodent chow) and water were provided *ad libitum*; animals were individually housed in all experiments starting 2 days before ICH to monitor food and water consumption, and to prevent fighting after surgery. Each single rat was considered an experimental unit.

### Experimental design

**Fig 1:**
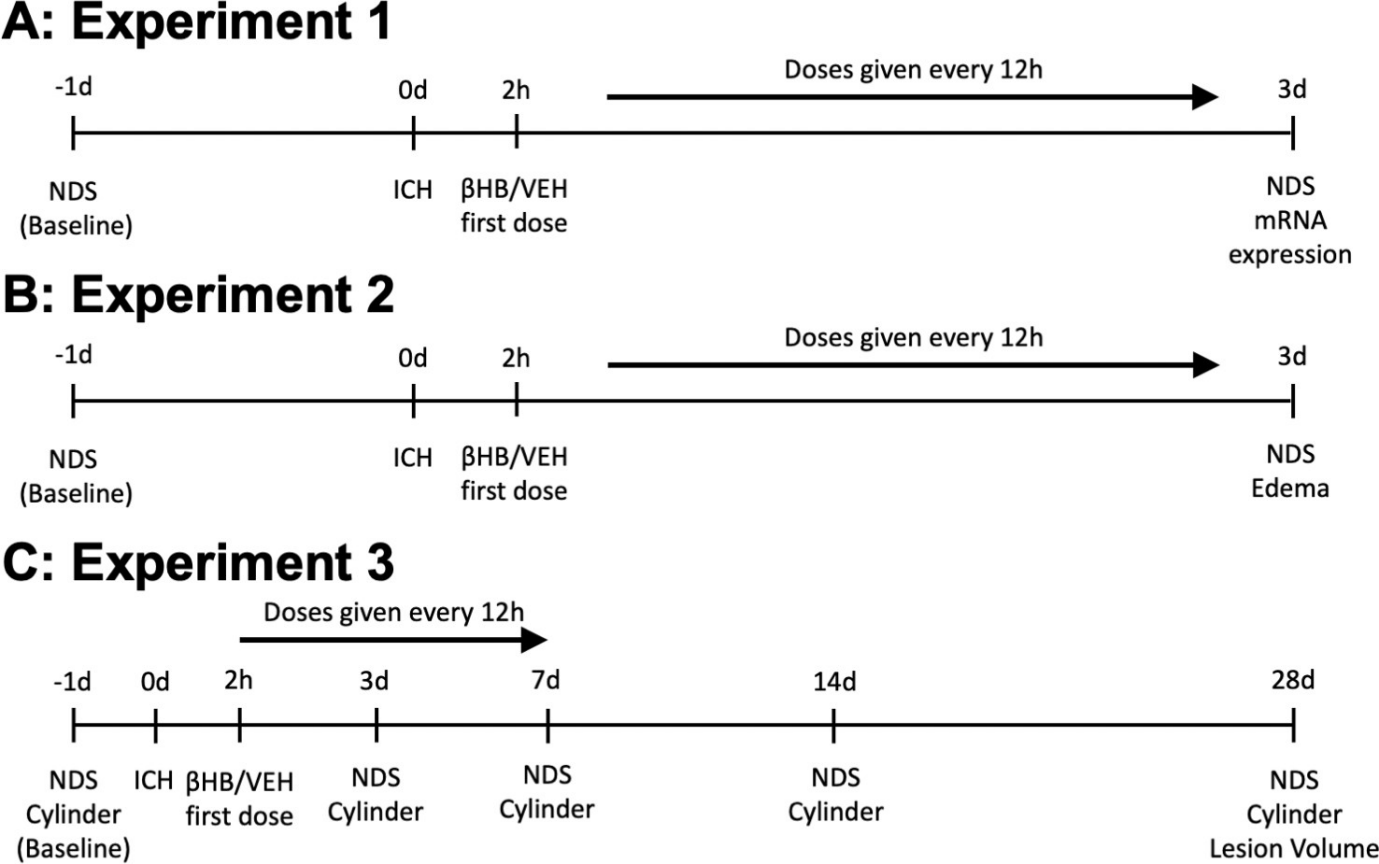
Experimental timelines. In experiments 1 (A) and 2 (B), rats were given either βHB or VEH starting 2 hours after ICH, and every 12 hours following until euthanasia 3 days post-ICH. Edema was the primary endpoint for experiment 2. C) Lesion volume was the primary endpoint in experiment 3, assessed 28 days after ICH. Rats were given either βHB or VEH for 7 days post-ICH. d: day(s); h: hour(s); NDS: neurological deficit scale; ICH: intracerebral hemorrhage; βHB: β-hydroxybutyrate.

To reduce stress associated with experimenter handling and treatment administration, all animals were familiarized to researchers prior to the start of behaviour training (experiment 1, two 10-minute sessions; experiments 2–3, three 10-minute sessions). All animals were trained on behavioural tests 2 days prior to induction of ICH, and baseline behavioural assessments were performed 1 day prior. Before all behavioural training and assessments, animals were left in the testing room to acclimatize for a period of at least 30 minutes. Rats were assigned to groups using a random number generator (random.org) immediately after ICH induction. Treatments were prepared by another researcher to ensure blinding. Researchers remained blinded to group allocation until all assessments were completed. Body weight, water intake, and food consumption were measured immediately prior to ICH and every 24 hours after ICH to monitor animal health. Experimental timelines are outlined in **Fig 1**.

#### Experiment 1

This experiment investigated the effects of βHB administration on inflammation and neurological deficits (Fig 1A). Rats were randomized to control VEH treatment or experimental βHB treatment groups (n = 8/group). Treatments were given for 3 days after ICH, and neurological deficits were assessed using the NDS at baseline and immediately prior to euthanasia (72 hours post-ICH). Brains were then quickly harvested for further analysis (transcript expression and cerebral βHB quantification). Experimental safety measures (i.e., body weight, food intake, and water consumption) were assessed daily.

#### Experiment 2

Edema was the primary endpoint for this experiment (Fig 1B). Rats were randomized into either the VEH or βHB groups (n = 12/groups) and given their respective treatments for days after ICH. Safety endpoints were measured daily. NDS assessments were performed at baseline and immediately prior to euthanasia (72 hours post-ICH). Edema was assessed at euthanasia (72 hours post-ICH).

#### Experiment 3

Lesion volume was our primary endpoint for this experiment (Fig 1C). Rats were randomized into either the VEH or βHB groups (n = 16/group). In this experiment, respective treatments were given for 7 days after ICH to potentially enhance the therapeutic effect of βHB, as cell death proceeds over weeks in this model [4, 35]. Behavioural endpoints (NDS and forelimb use asymmetry) were assessed at baseline, 3, 7, 14, and 28 days post-ICH. Lesion volume was quantified histologically at euthanasia (28 days post-ICH).

### Intracerebral hemorrhage

All animals underwent surgery to induce ICH, using the well-established collagenase model, as previously described [4, 30]. Rats were anaesthetized with isoflurane (4% induction, ∼2% maintenance in 60% N_2_O, remainder O_2_) prior to the initial scalp incision. A needle was placed into the left striatum, 6.5 mm below the skull surface through a drill hole (0.5 mm anterior, 3.5 mm lateral to Bregma). An ICH was induced by infusing 1.3 μL of bacterial collagenase (Type IV-S, Sigma, 0.6U/μL in saline); this dose was chosen to produce a 35–40 μL striatal bleed (a moderate-sized ICH) [36, 37]. Bupivacaine hydrochloride (0.5 mg/kg subcutaneous) was administered to the incision site before and after the surgery for pain relief. The hole was sealed using a small metal screw and the incision was closed using wound clips. Temperature was monitored and maintained at 37±0.5°C using a rectal temperature probe and a heated water blanket.

### Ketone treatment

Rats in the βHB group were given 250 mg/kg injections of racemic Na^+^-βHB (Sigma-Aldrich, 54965) in sterile isotonic saline (0.2g/mL subcutaneous, b.i.d. at 12-hour intervals) starting 2 hours after ICH induction. Only the predominant D-βHB can be catabolized for energy, but both D-βHB and L-βHB have signaling effects [38]. Thus, we used a racemic mixture to give the most reasonable increase in ketones while providing the metabolic value of D-βHB and the signaling effects of both enantiomers. Although our dose is higher than commonly used in most animal models of ischemic stroke and TBI, similar doses have been used in rats to provide neuroprotection in retinal ganglion cells and reduce hippocampal inflammation in response to chronic stress [32, 33]. Using allometric scaling [39], this dose is equivalent to ∼40 mg/kg in humans (2.8 g βHB for a 70 kg person); clinical studies show intravenous infusions of up to 100 g given over a few hours to be well-tolerated [40]. Treatment was delayed to 2 hours post-ICH to match the clinical feasibility of starting ketone therapy after ICH. Injections were all performed in the same room, and a dim red light was used to illuminate the room for no longer than 10 minutes for injections performed during the dark phase. Rats in the VEH group were given an equivalent volume of isotonic saline. Rats were euthanized 10 hours after the final subcutaneous injection.

### Neurological Deficit Scale (NDS)

All animals were trained and tested using the NDS to measure neurological deficits. This assessment generates a score based on the completion of 5 measures: spontaneous circling in a clear plexiglass cylinder, bilateral forepaw grasping ability, beam walking, hindlimb retraction, and bilateral forepaw flexion. Each task was scored from 0–3, except for hindlimb retraction which was scored from 0–2. Combination of these scores gives a total score between 0 (no deficits) and 14 (most severe deficits), and it has moderate predictive relationship to lesion volume [41]. Baseline assessments were performed 1 day prior to ICH surgery. Subsequent assessments were performed at 3 days after ICH (all experiments), and at 7, 14, and 28 days (experiment 3).

### Forelimb use asymmetry (cylinder)

The cylinder test was used to assess spontaneous forelimb use asymmetry as a secondary measure of functional outcome in experiment 3. This test modestly correlates with the volume of injury [41], and is a widely-used indicator of neurological impairments in models of stroke, including ICH. Animals were placed in a clear plexiglass cylinder for 5 minutes and videotaped from below. Each instance of forelimb use during push-offs, wall exploration, and landings was recorded and the limb used (contralateral or ipsilateral to injury) was noted. Push-offs are classified as the use of one or both forelimbs during rearing movements. The independent initial placement and subsequent contacts of either limb with the plexiglass cylinder wall is considered an instance of wall exploration. A landing is the use of one or both forelimbs to land after rearing. Independent forelimb use will be expressed as:

$$\frac{\text{number}\;\text{of}\;\text{contacts}\;\text{with}\;\text{contralateral}\;\text{limb}+\frac{1}{2}\text{number}\;\text{of}\;\text{contacts}\;\text{with}\;\text{both}}{\text{ipsilateral}\;\text{limb}\;\text{use}+\text{contralateral}\;\text{limb}\;\text{use}+\text{both}}\times 100.$$

Animals that failed to make 6 independent wall touches were excluded from analysis, as fewer touches are not considered sufficiently reliable to measure forelimb use frequency [41].

### Quantitative real-time PCR

In experiment 1, rats were anesthetized with isoflurane (4% in 60% N_2_O, remainder O_2_) and brains were harvested and quickly (within 90 seconds) dissected from 2 mm anterior to 4 mm posterior to the site of collagenase infusion into the following regions: ipsilateral striatum (containing the hematoma), ipsilateral cortex, contralateral striatum, contralateral cortex. All tissues were flash frozen in liquid nitrogen and stored at -80°C. Total RNA was extracted from tissue samples using TRIzol reagent (Invitrogen®) according to manufacturer’s instructions. RNA was quantified by measuring the absorbance at 260 nm (Nanodrop 2000, Thermo Fisher). Subsequently, first-strand cDNA synthesis was performed using 5X All-In-One RT MasterMix, according to the manufacturer’s instructions (Applied Biological Materials, G592). Quantitative real-time polymerase chain reaction (qPCR) for tissue samples from both the contralateral and ipsilateral striatum, as previously described [42, 43]. Quantification of transcript expression was performed by qPCR LightCycler® 480 multi(384)-well white reaction plates with PowerUP SYBR Green Master Mix (Applied Biosystems, A25742) in the LightCycler® 480 system (Roche Life Science). Primer sequences (Integrated DNA technologies, Coralville, IA) are shown in **Table 1**. The qPCR data was analyzed using the relative gene expression (ΔΔCt) method with β-actin as the housekeeping gene.

**Table 1:** Rat primer sequences used for quantitative real-time-PCR.

Gene	Forward Primer	Reverse Primer
*Actb*	**AACCCTAAGGCCAACCGTG**	**TACGTACATGGCTGGGGTGT**
*Cx3c11*	**GCCATCGGTGCAATCTATCT**	**GCACCCAAACCGAAGTCATA**
*Il1b*	**CACCTCTCAAGCAGAGCACAG**	**CCAGGGCTCTGTTCATTG**
*Mcp1*	**CTTCTGGGCCTGTTGTTCA**	**TTCTTTGGGACACCTGCTG**
*Tnfa*	**CCACCAGTTGGTTGTCTTTG**	**CCACCACGCTCTTCTGTCTA**

CX3CL1: C-X3-C motif chemokine ligand 1; IL1B: interleukin 1-beta; MCP1: monocyte chemoattractant protein-1; TNFA: tumor necrosis factor alpha.

We quantified the expression of the following genes: *Cx3cl1*, *Mcp1*, *Il1b*, and *Tnfa*. *Cx3cl1* encodes C-X3-C motif chemokine ligand 1 (CX3CL11), and *Mcp1* (also known as *Ccl2*) encodes monocyte chemoattractant protein-1 (MCP1). Both are chemokines produced by neurons that induce microglial and leukocyte chemotaxis to the perihematomal area after ICH [44, 45]. *Il1b* encodes IL-1β, which is primarily an NLRP3 inflammasome-dependent pro-inflammatory cytokine, and is associated with worsened injury and outcome in ICH [17]. *Tnfa* encodes TNFα, another pro-inflammatory cytokine that can activate the NLRP3 inflammasome and contributes to inflammatory injury in ICH [46–48]. Together, expression of these transcripts provide an indication of the regulation of important inflammatory parameters that contribute to secondary injury after ICH.

### Brain β-hydroxybutyrate levels

In experiment 1, the posterior brain (all tissue from 4 mm posterior of the collagenase infusion site to the cerebellum) was collected, flash frozen in liquid nitrogen, and stored at -80°C. Concentrations of βHB in the posterior brain were assessed using a colorimetric assay kit (Cayman Chemical, item no. 700190). This assay was performed according to the manufacturer’s instructions, and plates were read using a spectrophotometer (Synergy H4 Hybrid Microplate Reader, BioTek) at an absorbance of 450 nm, as suggested by the manufacturer.

### Edema

Animals in experiment 2 were anaesthetized with isoflurane (4% in 60% N_2_O, remainder O_2_) at 72 hours after ICH, and quickly decapitated. Brains were harvested and samples were taken from 2 mm anterior and 4 mm posterior to the site of collagenase infusion, and further dissected to isolate the striatum and cortex from both hemispheres. Cerebellum tissue was also taken as a control. Tissue samples were weighed immediately before and after baking at 100°C for 24 hours. Edema was calculated as brain water content using the following formula, as done previously [36, 49]:

$$\frac{\text{wet}\;\text{weight}-\text{dry}\;\text{weight}}{\text{wet}\;\text{weight}}\times 100.$$

### Histology (lesion volume)

Animals in experiment 3 were euthanized 28 days after ICH using a lethal injection of sodium pentobarbital (100 mg/kg IP, Bimeda) and transcardially perfused with 0.9% saline, followed by 10% neutral buffered formalin. Brains were harvested and post-fixed in formalin before being sliced into 80 μm-thick coronal sections using a vibratome (Leica VT1200 S). Sections were taken every 320 μm (1 in every 4 sections) and stained with cresyl violet for lesion volume analysis; the area of sectioning encompassed the entire lesion. Lesion volume was determined by manually calculating the total tissue lost using ImageJ, as done previously [36, 41]. Briefly, the volume of remaining tissue in the ipsilateral hemisphere was subtracted from the volume of tissue in the intact hemisphere, accounting for atrophy and cavity formation. Total volume of each hemisphere is calculated as follows:

average(totalareaofhemisphere−areaofventricle−areaofdamage)×intervalbetweensections×numberofsections.

### Statistical analysis

Sample sizes were determined *a priori* using G*Power (Universität Düsseldorf, v3.1.9) based on previous work and/or sample size calculations (two-tailed tests at α = 0.05, power of ≥80%). For experiment 1, we determined 8 rats/group to be sufficient based on historical transcript expression data and anticipated variability with the collagenase model of ICH [36, 42, 50]. For experiment 2, we calculated that group sizes of 12 rats/group would have 87% power to detect a 1% change in brain water content based on a standard deviation (SD) of 0.75% [37]. For experiment 3, 16 rats/group were needed to have 81% power to detect a 40% change in lesion volume (SD = 13.47 μL) [37].

All data were analyzed using GraphPad Prism (v 6.0, GraphPad Software Inc, La Jolla, CA). Data are presented as mean ± 95% confidence interval (CI) or median ± interquartile range (IQR) as indicated in figures. Body weight and water consumption data (experiment 1 and 2) were analyzed using a repeated measures ANOVA. This test was also used for food consumption data in experiment 2; a mixed-effects analysis was required for food consumption in experiment 1 due to 1 missing data point. Tukey’s multiple comparisons test was used post-hoc for body weight, and food and water consumption data to compare treatment groups on each day and to compare each time assessed. Striatal transcript expression (qPCR) data were analyzed and presented relative to the contralateral striatum of the VEH group (experiment 1); comparisons were made using a two-way ANOVA, and Sidak’s multiple comparisons test was used to determine differences between groups (βHB vs VEH) and brain regions (ipsilateral vs contralateral striatum). Cerebral βHB level (experiment 1), brain water content (experiment 2), and lesion volume data were analyzed using two-tailed independent sample t-tests. Mann-Whitney U tests were performed to compare ordinal NDS data between groups and one-sample Wilcoxon tests were used to compare NDS data between time points in all experiments. A mixed-effects analysis was used instead of a repeated measures ANOVA to analyze the asymmetry score on the cylinder test due to exclusions, and Tukey’s multiple comparisons test was planned as a post-hoc test. A significance level of α = 0.05 was used to determine significant differences.

## Results

### βHB treatment reduces perihematomal inflammation

**Fig 2:**
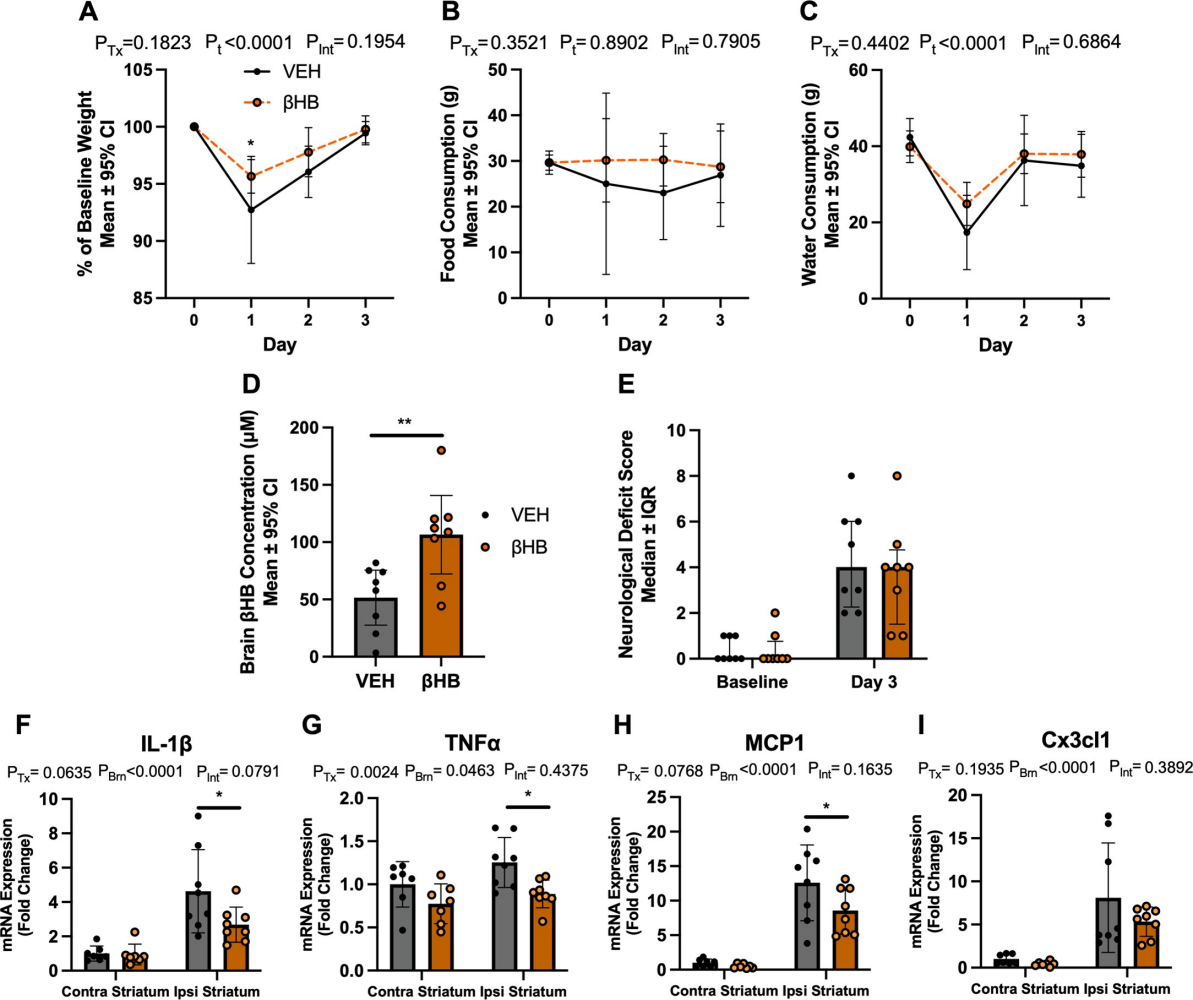
Initial assessment of βHB treatment effects. A) In experiment 1, body weight was reduced on day 1 after ICH (day 1, p<0.0001), and the βHB group weighed more than the VEH group on day 1 (p = 0.0250). Body weight was not affected by βHB at any other time point (all p≥0.1826). B) Food consumption was similar between VEH and βHB groups on all days measured before and after surgery (all p≥0.2327). C) Water consumption was decreased on day 1 (p<0.0001) and was not affected by treatment at all times assessed (all p≥0.0970). D) βHB administration increased βHB concentrations in the brain compared to VEH controls (p = 0.0077). E) Both groups had similar NDS scores at baseline. βHB did not affect NDS scores 3 days after ICH (p = 0.6232) in experiment 1. F) There was an increase of *Il1b* transcript expression in the ipsilateral striatum for both the VEH (p<0.0001) and βHB (p = 0.0481) groups. This increase was significantly reduced in the βHB group (p = 0.0210). G) Expression of *Tnfa* in the ipsilateral striatum was significantly reduced in the βHB group (p = 0.0108). H) There was an increase of *Mcp1* expression in the ipsilateral striatum for both the VEH (p<0.0001) and βHB (p = 0.0002) groups. This increase was reduced in the βHB group (p = 0.0473). I) There was an increase of *Cx3cl1* expression in the ipsilateral striatum for both the VEH (p = 0.0009) and βHB (p = 0.0202) groups. This increase was not significantly reduced in the βHB group (p = 0.2216). N = 7-8/group for all endpoints in experiment 1. Data are presented as mean ± 95% confidence interval (CI) or median ± interquartile range (IQR). CI: confidence interval; ICH: intracerebral hemorrhage; IQR: interquartile range; NDS: neurological deficit scale; P_Tx_: p-value of main treatment effect; P_t_: p-value of main time effect; P_Brn_: p-value of main brain area effect (contra vs. ipsi striatum; P_Int_: p-value of interaction; βHB: β-hydroxybutyrate. *: p<0.05; **: p<0.01.

This experiment used 17 rats. Prior to being assigned to a treatment group, 1 rat died from a surgical error, leaving group sizes of n = 8/group. As expected, ICH reduced body weight after injury (**Fig 2A**; p<0.0001) and the βHB group weighed more than the VEH group on day 1 after ICH (p = 0.0250). No difference in body weight was found between treatment groups at all other times assessed (all p≥0.1826). Food consumption was similar between VEH and βHB groups on all days measured before and after surgery (**Fig 2B**; all p≥0.2327). Water consumption was reduced after ICH (**Fig 2C**; p<0.0001) but was not different between groups at all times assessed (all p≥0.0970). There was a 2-fold increase of βHB concentration in the posterior brain (**Fig 2D**; mean difference 54.91 (± 37.91 95% CI) μM; p = 0.0077) of rats given βHB. There was a median 4-point increase in NDS scores (**Fig 2E**; p<0.0001) 3 days post-ICH than at baseline for both groups, and no differences in NDS scores between groups at 3 days post-ICH (p = 0.6232). Thus, ICH caused an impairment that was not affected by βHB treatment.

In assessing inflammatory transcripts, one sample per group from the contralateral striatum was excluded due to RNA extraction and cDNA synthesis errors. This left n = 7/group for contralateral striatum samples, and n = 8/group for ipsilateral striatum samples. Compared to the contralateral striatum, transcript expression of *Il1b* (**Fig 2F**), *Mcp1* (**Fig 2H**), and *Cx3cl1* (**Fig 2I**) were increased in the ipsilateral striatum for both VEH (p<0.0001, p<0.0001, and p = 0.0009, respectively) and βHB (p = 0.0481, p = 0.0002, and p = 0.0202, respectively) treatment groups, indicating that a local inflammatory response was observed at 3 days post-ICH in the target area but not in the contralateral striatum. Importantly, the expression of *Il1b* (**Fig 2F**), *Tnfa* (**Fig 2G**), and *Mcp1* (**Fig 2H**) in the injured ipsilateral striatum were reduced (p = 0.0210, p = 0.0108, and p = 0.0473, respectively) in the βHB-treated group. Expression of *Cx3cl1* in the ipsilateral striatum was not different between groups (p = 0.2216).

### βHB treatment does not reduce perihematomal edema despite improving NDS 3 days post-ICH

**Fig 3:**
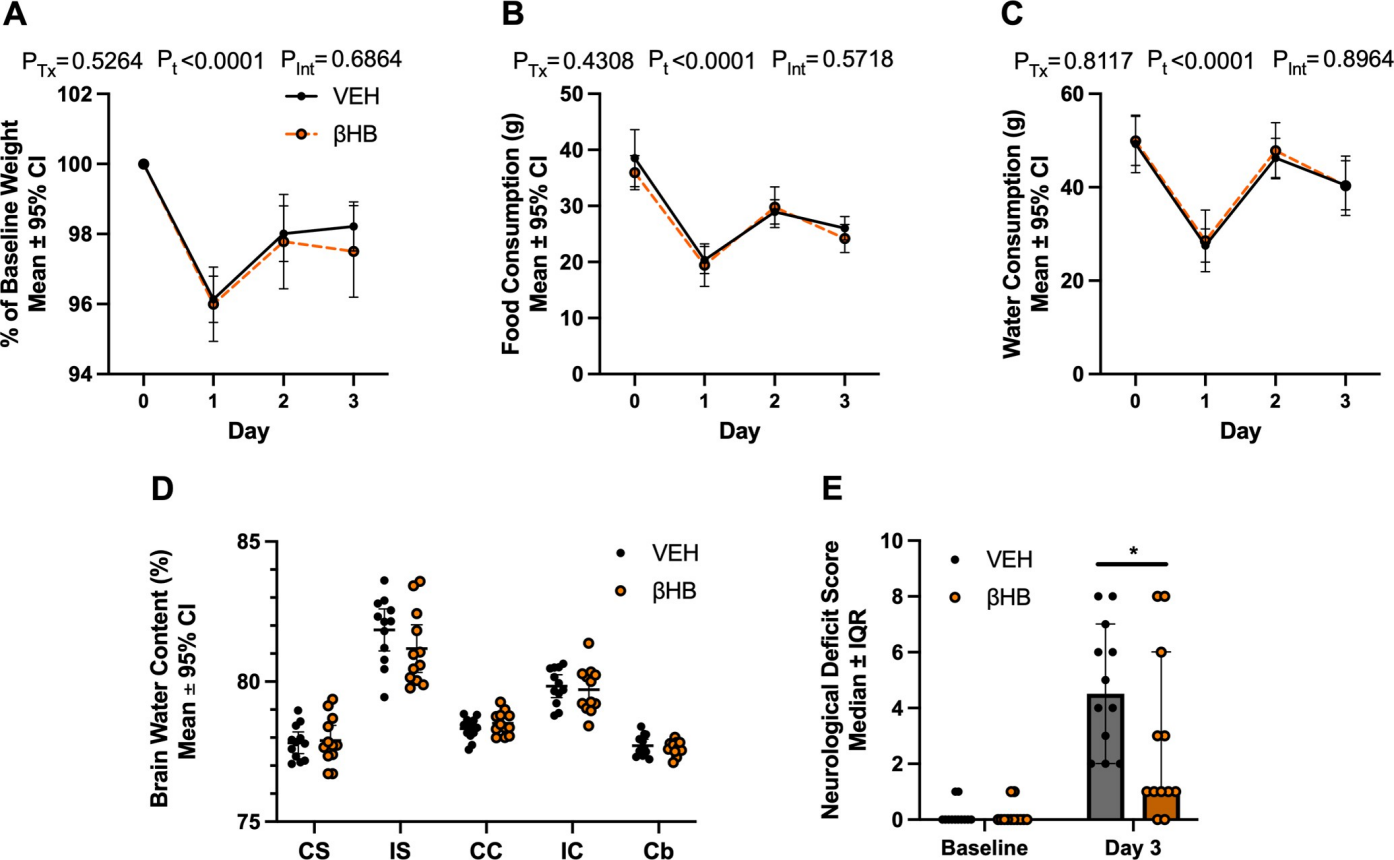
Effects of βHB treatment on edema and NDS 3 days after ICH. A) In experiment 2, body weight was decreased on day 1 after ICH (p<0.0001), but not different between treatment groups at all times assessed (all p≥0.2118). B) Food consumption was decreased on day 1 following injury (p<0.0001), but not affected by treatment (all p≥0.2205). C) Water consumption was reduced on day 1 (p<0.0001) but was not affected by treatment (all p≥0.6566). D) All animals had higher brain water content in the ipsilateral striatum compared to the contralateral striatum. There were no significant treatment effects on brain water content in any regions (all p≥0.2110). E) Both groups had similar NDS scores at baseline. Significant neurological deficits were observed in both treatment groups 3 days after ICH (p<0.0001). NDS scores were significantly improved in the βHB group (p = 0.0416) in experiment 2. N = 12/group for all endpoints in experiment 2. Data are presented as mean ± 95% confidence interval (CI) or median ± interquartile range (IQR). CI: confidence interval; Cb: cerebellum; CC: contralateral cortex; CS: contralateral striatum; IC: ipsilateral cortex; IS: ipsilateral striatum; NDS: neurological deficit scale; P_Tx_: p-value of main treatment effect; P_t_: p-value of main time effect; P_Int_: p-value of interaction; IQR: interquartile range; βHB: β-hydroxybutyrate. *: p<0.05.

This experiment used 24 rats. There were no exclusions or unexpected mortality in this experiment, leaving group sizes of n = 12/group. The ICH reduced body weight on day 1 after injury (**Fig 3A**; p<0.0001) and no difference in body weight was found between groups at all times assessed (all p≥0.2118). Food and water consumption were also reduced on day 1 following ICH (**Fig 3B**, C; both p<0.0001) and there were no differences between treatment groups at all times assessed (all p≥0.2205). Collagenase infusion increased brain water content in the injured striatum (**Fig 3D**; p<0.0001), by 4.0 ± 0.8% for VEH group and 3.3 ± 0.95% for βHB group, when compared to the intact contralateral striatum. There were no treatment effects on brain water content in any brain region (**Fig 3D**; all p≥0.2110). Compared to baseline, NDS scores were greater in both groups by a median 3 points at 3 days post-ICH indicating neurological deficits from the ICH (**Fig 3E**; p<0.0001). However, these deficits in NDS scores were improved in the βHB-treated group compared to VEH (**Fig 3E**, 3.5-point median difference, p = 0.0416).

### 7 days of βHB treatment post-ICH does not chronically improve lesion volume

A total of 32 rats were used for this experiment. There was no unexpected mortality, leaving n = 16 rats/group. Data from trials in which animals failed to make 6 independent wall touches were excluded from forelimb use asymmetry analysis (n = 2 in VEH group and 5 in βHB group on days 3 and 7, n = 2 in βHB group on day 14, n = 1 in VEH group and n = 1 in βHB group on day 28). For lesion volume analysis, 1 animal in the VEH group and 2 in the βHB group were excluded due to histological sectioning errors during tissue processing.

**Fig 4:**
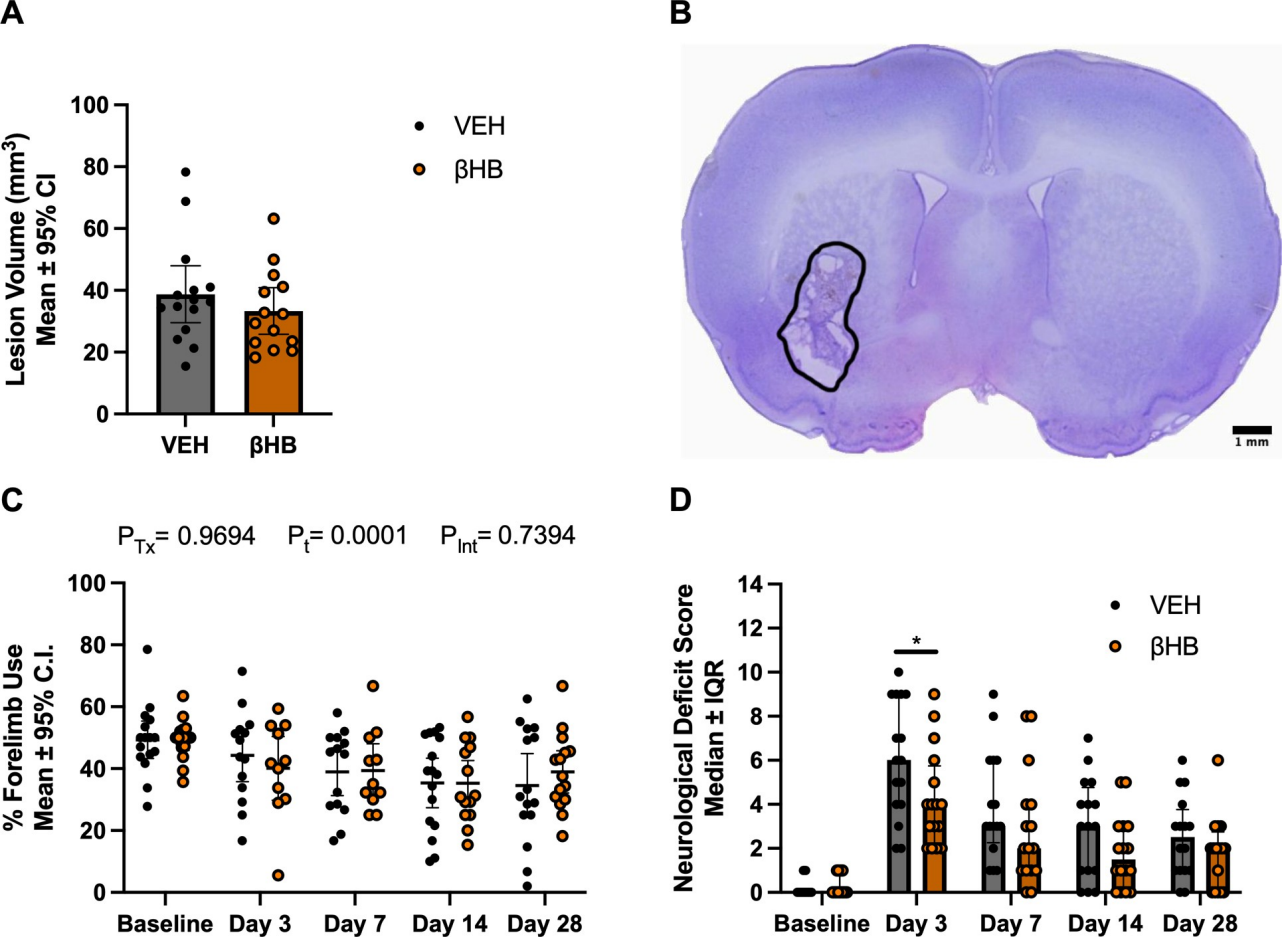
Long-term outcomes of 7-day βHB treatment. A) In experiment 3, lesion volume at 28 days after ICH was slightly less in rats treated with βHB, but this effect was not significant (p = 0.3381). B) Representative lesion volume image (βHB group) taken 28 days post-ICH, representing area of dead tissue (within solid line). Scale bar represents 1 mm. C) No differences were seen on the forelimb use asymmetry test at any time assessed (all p≥0.4511). D) While rats in the βHB group performed better on the NDS at 3 days post ICH compared to the VEH group (p = 0.0459), no differences were detected between groups at any other time assessed (all p≥0.1546). Data are presented as mean ± 95% confidence interval (CI) or median ± interquartile range (IQR). CI: confidence interval; NDS: neurological deficit scale; IQR: interquartile range; P_Tx_: p-value of main treatment effect; P_t_: p-value of main time effect; P_Int_: p-value of interaction; βHB: β-hydroxybutyrate. *: p<0.05.

The collagenase model resulted in an average lesion volume of 36.1 mm^3^, which is consistent with previous results [51]. There were no differences in total tissue lost 28 days post-ICH (**Fig 4A**; p = 0.3381). However, at 7, 14, and 28 days post-ICH, contralateral forelimb use analysis revealed a significant time effect compared to baseline (**Fig 4C**; p = 0.0087, p = 0.0013, p = 0.0062, respectively), showing that ICH resulted in sustained neurological deficits to forelimb use. There were no differences in forelimb use between treatment groups at any times (all p≥0.4511). Neurological deficits were evident at all times assessed post-ICH compared to baseline assessment (**Fig 4D**; all p<0.0001), indicated by higher NDS scores. At 3 days post-ICH, these deficits were improved in the βHB group (2-point median difference) compared to VEH (p = 0.0459). No group differences were observed at any other times (all p≥0.1546).

### Pooled NDS scores show that βHB improves NDS 3 days post-ICH

**Fig 5:**
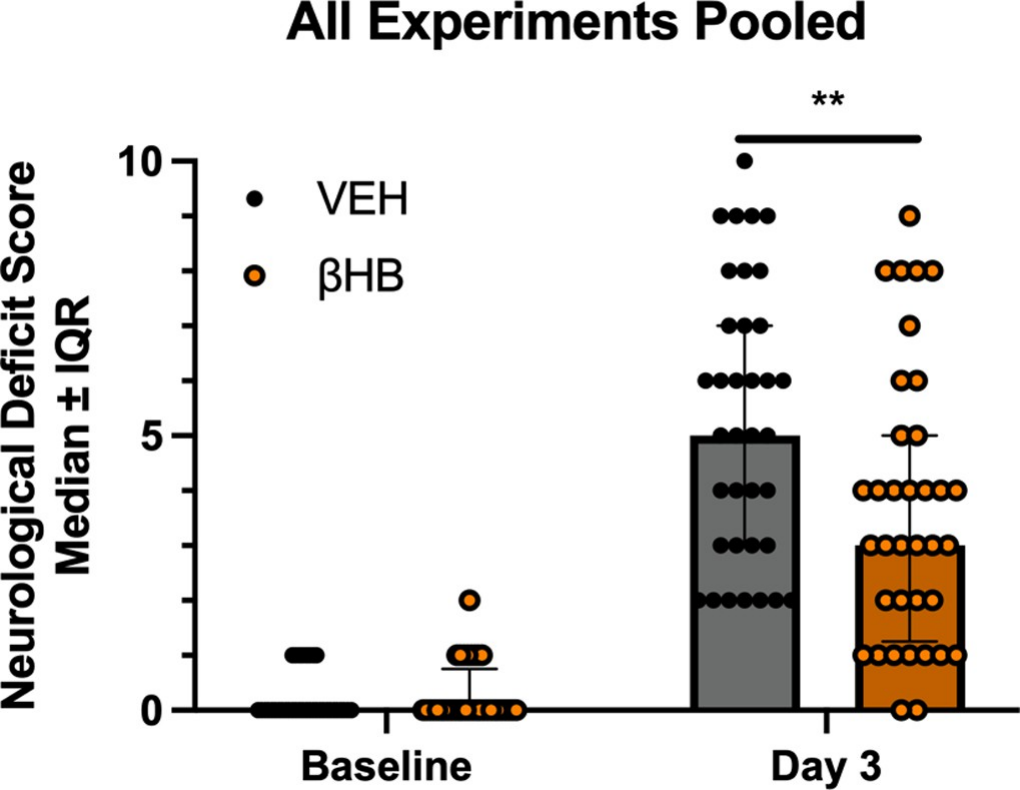
Pooled neurological deficit scale scores. Across all experiments, there were significant neurological deficits at 3 days post-ICH compared to baseline. These deficits were significantly improved in the βHB-treated group (2-point difference, p = 0.0076) compared to the VEH-treated group at 3 days post-ICH. Data are presented as median ± interquartile range (IQR). **: p<0.01.

The NDS data collected on day 3 post-ICH from all experiments was pooled as a post-hoc analysis with a total of n = 36 rats/group. This analysis showed neurological deficits groups across all experiments (**Fig 5**; p<0.0001) at 3 days post-ICH compared to baseline. Notably, these deficits were significantly improved in the βHB-treated group (2-point difference, p = 0.0076) compared to VEH at 3 days post-ICH.

## Discussion

As a significant contributor of neurological injury and disability, ICH remains an important condition that requires new treatment approaches to improve these outcomes. Ketone therapy has provided significant neuroprotection in various models of brain injury, including ischemic stroke and TBI. As the primary ketone, βHB, has anti-inflammatory properties, we sought to investigate the therapeutic potential of exogenous βHB after the induction of an ICH. In this study, we present evidence that exogenous βHB treatment provides early functional benefits with a concurrent attenuation of inflammation, but not edema. Furthermore, we demonstrate that our treatment regimen safely and effectively increases βHB levels in the brain at a time of considerable inflammation, edema and cell death in this model. However, the early benefits of βHB treatment did not translate into lasting effects on brain injury or behavioural outcomes. As reductions in inflammation do not necessarily translate into improvements in injury or long-term outcome, this study demonstrates that the effects of βHB treatment on mechanisms of secondary brain injury may not produce lasting behavioural benefit after ICH. Nevertheless, the early benefits shown here suggest that other dosing regimens or combinations with co-therapies (e.g., rehabilitation [52]) might lead to greater and more profound benefits over a longer term.

In our first set of experiments, we demonstrate that βHB treatment reduced markers of inflammation around the injury acutely after ICH. Inflammatory processes are pronounced in the collagenase model of ICH [31], which we confirmed by the observed increase in transcript expression of the pro-inflammatory *Il1b*, *Mcp1*, and *Cx3cl1* within the injured striatum 3 days post-ICH. The βHB treatment approximately doubled βHB concentrations in the brain and reduced transcript expression of *Il1b*, *Tnfα*, and *Mcp1* in the injured striatum by 42–59% relative to the contralateral striatum of the VEH group 3 days post-ICH. While protein levels may differ from gene expression level, the expression of these transcripts provides an indication of the regulation of important inflammatory processes. Our results indicate that βHB bypassed the BBB and reduced pro-inflammatory processes around the injury. Generally, downregulation and inhibition of pro-inflammatory cytokines (i.e., IL-1β, TNFα) has been shown to reduce edema, brain damage, and neurological deficits after ICH in rodent and porcine models [46–48, 53]. As for βHB, its anti-inflammatory effects are primarily thought to be due to inhibiting the NLRP3 inflammasome, which promotes IL-1β production [21]. Thus, the observed reduction in transcript expression of *Il-1β* suggest that our βHB treatment may inhibit the NLRP3 inflammasome around the injury after ICH. Importantly, βHB treatment was well-tolerated as it did not affect our safety parameters (i.e., body weight and food or water consumption). Further studies should use additional approaches, such as different endpoints and times, and assess a comprehensive perihematomal inflammatory profile to better understand inflammatory changes in the brain (e.g., microglial activation, monocyte infiltration, etc.) attributed to ketone therapy after ICH. This includes evaluating the time course of anti-inflammatory effects and experimentation to prove a linkage between those effects and any functional or histological benefits.

In addition to anti-inflammatory effects, βHB treatment provided early functional benefits, as measured by improvements in NDS scores 3 days post-ICH. Although the first experiment did not show this effect, the subsequent experiments and pooled analysis across all experiments suggest show that βHB treatment resulted in clear functional improvements by ∼2 points on the NDS assessment early after ICH. While the reason for this discrepancy is unclear, the lower sample size in experiment 1 (n = 8/group) increases the probability that this experiment missed a true effect. The pooled analysis across all experiments (n = 36/group) strongly indicates that functional improvements measured by NDS are present early after ICH. However, there were no benefits observed using the cylinder test of forelimb use asymmetry in experiment 3, indicating that the early functional benefits of βHB treatment may not include improvements in spontaneous contralateral forelimb use. Nevertheless, additional tests with greater sensitivity should be considered for future investigation.

Although the observed reductions in inflammation and early functional improvements are promising, such early assessments are not sufficient for one to assume that there would be persistent benefits. Instead, long-term outcomes are more clinically important where the main objective of patient care is to persistently improve outcomes, such as activities of daily living and quality of life [54]. Both NDS and forelimb use assessments revealed no functional benefit in later assessments up to 28 days after ICH. While this may suggest that the early behavioral benefits conferred by βHB treatment after ICH may not persist into the long-term, both the NDS and the forelimb use test are most sensitive early after ICH [41]. Thus, other behavioural tests (e.g., the staircase test or the adhesive removal test) might have better detected long-term functional differences after ICH [41]. Furthermore, ketones also can have various other actions, such as altering blood flow or cerebral metabolism [55], which may have had short-lived and transient symptomatic benefits that disappeared after treatment cessation. Thus, there is a need to consider more protracted dosing in future studies.

The lack of the lasting functional benefit may also be explained by our findings regarding brain edema (brain water content) and brain injury size (lesion volume). Despite the anti-inflammatory effects, we observed no differences in edema between the βHB and VEH groups. It was important to measure edema to assess whether the anti-inflammatory effect was sufficient to significantly reduce a strong indicator of outcome and secondary injury [11]. The absence of benefit on edema is intriguing, as various approaches to ketone therapy have reduced edema in animal models of other brain injuries [22, 26, 56, 57]. However, these studies use preventative approaches that begin before the onset of brain injury, whereas we used a βHB treatment after the ICH was established. Even if benefit was found with a treatment starting before injury, this would not be meaningful to most patients, as they would not start treatments until after the initial insult. Thus, we administered βHB starting 2 hours after ICH to be more clinically relevant. Given that there were no significant treatment effects on edema (a strong indicator of secondary injury) it is not surprising that we also did not find any significant benefits of βHB on brain injury 28 days after ICH. These long-term findings indicate the βHB treatment regimen used did not provide significant neuroprotective benefits after ICH, which may explain why the treatment did not provide lasting functional benefits. Such long-term assessments are underutilized in ICH research; only 6% of all studies assess injury size and 18.5% conduct functional assessments more than 2 weeks after ICH [10]. Despite the negative findings here, our long-term assessment contributes valuable data that establish a strong precedent for future studies of ketone therapy in ICH to obtain long-term findings to support their conclusions.

As the effects of ketone therapy after brain injury will be affected by the dose of βHB given [28], other dosing regimens may yield more desirable effects on inflammation, edema, brain injury size, and ultimately, functional outcomes. Our study gave 250 mg/kg of βHB (subcutaneous), which is a higher dose than that used in most animal models of TBI and focal ischemia. However, comparing doses across studies is difficult owing to heterogeneity in ketone therapy (e.g., ketogenic diet, fasting, ketone ester, ketone salt), dosing frequency and route of administration. Difficulties exist comparing our observed elevation of ketone levels to existing research, as most studies only assess systemic (e.g., plasma, serum) levels of ketones, often in the context of ketogenic diets. Because of the difficulty comparing ketone therapy regimens, future work should investigate alternate doses and better characterize ketone distribution after ICH.

Our results provide important data that will guide future studies investigating ketone therapy after ICH. Given the early benefits of βHB treatment on inflammation and functional outcomes and the non-significant trends towards improvement for both edema and lesion volume, future studies should investigate ketone therapy after ICH with higher statistical power to detect smaller effect sizes that may have been missed in our study, but that may nonetheless be clinically important. Future work should also closely investigate other doses and administration routes to optimize the effects on inflammation and to further assess mechanisms of ketones, such as antioxidant activity, vascular and metabolic effects, amongst others [19]. Other therapies (e.g., rehabilitation [52]) used in conjunction with βHB should be investigated, as they may have an additive effect on secondary mechanisms of injury.

In conclusion, our findings demonstrate that exogenous ketone therapy does not provide lasting behavioural benefits after ICH. Although we found βHB treatment to reduce markers of inflammation and provide some early behavioural improvements, no clear evidence of lasting benefit was observed. Despite our negative long-term findings, the early benefits observed are promising and suggest that other dosing regimens and methods of ketone elevation may lead to greater long-term benefits and should be investigated to gain a fulsome understanding of ketone therapy in ICH.

## Supporting information

S1 Dataset Original data. (XLSX)
